# Neonatal Bladder Inflammation Results in Adult Female Mouse Phenotype With Increased Frequency and Nociceptive Responses to Bladder Filling

**DOI:** 10.3389/fnsys.2022.858220

**Published:** 2022-03-11

**Authors:** Buffie Clodfelder-Miller, Timothy J. Ness, Jennifer J. DeBerry

**Affiliations:** Department of Anesthesiology and Perioperative Medicine, University of Alabama at Birmingham, Birmingham, AL, United States

**Keywords:** interstitial cystitis/bladder pain syndrome, visceral pain, developmental, neonatal, inflammation

## Abstract

Bladder pain and hypersensitivity to bladder filling are clinically common, but animal models examining syndromes with these features are limited. A rat model of bladder hypersensitivity produced by neonatal bladder inflammation (NBI) has been reported to have many of the clinical features of bladder pain syndromes. The present study sought to determine whether similar hypersensitivity might be induced by NBI in mice. Female C57BL6/J mice had NBI induced on postnatal days P12-14 by the intravesical administration of zymosan. As adults (12–14 weeks of age), the mice were examined for hypersensitivity of their bladders as: spontaneous voiding and evoked cystometrograms at baseline, and visceromotor responses (VMRs) to urinary bladder distension (UBD) following a secondary insult (either repeated bladder inflammation or acute stress induced by footshock). Mice that experienced NBI demonstrated hypersensitivity, when compared with control mice, manifested as increased spontaneous voiding, increased frequency of evoked voids during intravesical saline infusion, and increased vigor of VMRs to UBD following either acute bladder inflammation or acute stress. This recapitulates the hallmark features of clinical painful bladder disorders and suggest utility of this murine model for the study of these disorders while allowing methodological expansion into well-established genetic and immunological models.

## Highlights

- The present study demonstrates that neonatal bladder inflammation in female mice results in an adult phenotype which has increased urinary frequency and augmented responses to bladder filling.
- These are hallmark features of human painful bladder disorders and should allow methodological expansion of these disorders into well-established murine genetic/immunologic models.

## Introduction

Interstitial cystitis/bladder pain syndrome (IC/BPS) is a disorder characterized by urinary frequency and pelvic pain with bladder filling (Bogart and Clemens, 2007; Clemens et al., 2019). The etiology of IC/BPS remains elusive, treatment options are limited, and to date, there is no cure. Animal models of bladder pain have been developed which mimic some of the features of IC/BPS (Lai et al., 2015) but each has been noted to have limitations. We have developed an animal model in the rat that recapitulates many “clinical” features that are observed in patients with IC/BPS including the following (Randich et al., 2006, 2009; Deberry et al., 2010; Ness et al., 2021): (1) “flares” of augmented nociceptive responses to urinary bladder distension (UBD) following sensitizing events; (2) increased voiding frequency; (3) decreased cystometric pressure/volume thresholds for activating micturition responses; (4) nociceptive responses to intravesical solutions such as potassium chloride; (5) increased pelvic floor muscular tone; (6) evidence of submucosal hemorrhages following sustained hydrodistension and (7) increased cold sensitivity of the bladder. What produced these alterations in phenotype in adult rats was the experience of neonatal bladder inflammation (NBI) during a critical period of development on postnatal days 14 through 16 (P14-P16). This is the time when inhibitory systems descending from brainstem structures are developing in rat pups (Fitzgerald, 2005). NBI is a correlate to childhood bladder infections in humans. Epidemiological data suggests increased bladder infections and need for antibiotic therapy during childhood in adult individuals with IC/BPS (Peters et al., 2009) which is additional evidence that NBI has correlative if not causative relevance to the pathophysiology of IC/BPS. It is notable that in the absence of a second, adult “insult” such as re-inflammation of the bladder, there are limited differences in nociceptive responses to bladder distension, and histologically the bladders appear normal (Deberry et al., 2010). However, upon the introduction of a second “insult,” exaggerated nociceptive responses to UBD occur in rats that experienced NBI when challenged as adults with one of the following (Ness et al., 2021): acute bladder inflammation (ABI) induced by intravesical administration of either zymosan or lipopolysaccharide (to mimic a yeast and/or bacterial infection, respectively); exposure to an acute stressor (footshock); inflammation of a non-bladder structure with afferent input to the same spinal segments as bladder afferents (footpad injections); and heterosegmental neuropathic inputs (trigeminal nerve ligations). NBI also leads to alterations in bladder/spinal cord neuropeptide content (Shaffer et al., 2011), responses to opioid agonists/antagonists (Deberry et al., 2007; Shaffer et al., 2013a,b), increased neurogenic inflammation of the bladder in response to intravesical TRPA1 agonists (Deberry et al., 2010), altered responsiveness to neuromodulatory manipulations (Ness et al., 2016), altered spinal neuronal responses to visceral stimuli (Ness and Randich, 2010; Miranda et al., 2011; Kannampalli et al., 2017) and altered GABA-A, KCC2 and VGAT mRNA expression at spinal levels (Sengupta et al., 2013; Zhang et al., 2017). Despite the extensive nature of these studies, the precise neurophysiology and neurochemistry leading to exaggerated nociceptive responses are still yet-to-be-determined.

Although useful, there are clear methodological limitations associated with the use of rat models. Use of mice for the study of other painful disorders has allowed for expansion into use of numerous genetic and immunological murine models with selective alteration of reactive systems (Mogil, 2012, 2019). Cardiovascular, cystometric and visceromotor responses (VMRs) to UBD have been characterized in mice and demonstrated to have experimental utility in association with painful bladder disorders (Ness and Elhefni, 2004; Sadler et al., 2013; Lai et al., 2015; Fuentes and Christianson, 2018) but in those studies the animal subjects lack many of the clinical features of IC/BPS. Given the clinically significant results noted above which have been observed in rats that experienced NBI, the present investigation sought to characterize the effects of the same manipulation in mice, hypothesizing that they would be similar to those noted in rats. Since urinary frequency and pain with UBD are the hallmark features of IC/BPS (Hand, 1949; Koziol et al., 1993; Jones and Nyberg, 1997; Bogart and Clemens, 2007; Clemens et al., 2019) and of the NBI rat model, the initial characterization of murine responses following NBI comprised measures of micturition and nociceptive responses to UBD.

## Materials and Methods

### General

All protocols were approved by the Institutional Animal Care and Use Committee at the University of Alabama at Birmingham and conformed to NIH guidelines for the care and use of laboratory animals. All mice were C57/BL6 mice raised from birth in colonies maintained at the University of Alabama at Birmingham with original sourcing from Jackson Laboratories (Bar Harbor, Maine). All mice had food and water available on an *ad libitum* basis except where specifically noted. Mice were housed in standard polycarbonate cages with woodchip bedding and nestlets. A 12:12 h light-dark cycle was maintained. All mice received neonatal treatments (described below) and subsequent assays were performed as adults (12–14 weeks of age). Voiding and cystometric measures were performed without any additional treatments. VMRs (described below) were elicited after secondary adult treatments consisting of either adult bladder inflammation (ABI) or acute footshock stress (AFS).

### Neonatal Bladder Inflammation

A total of 85 female pups were raised from birth for these studies. Beginning on postnatal day 12 (P12), pups were isoflurane-anesthetized (5% induction, 2.5% maintenance) *via* mask, had their urethras swabbed with iodine-povidone solution, and were kept warm with an isothermal pad. All mice were given ampicillin (50 mg/kg, s.c.). NBI-treated mice had PE-10 (polyethylene tubing, Intramedic cat#427401) placed transurethrally and received intravesical administration of approximately 50 μl of zymosan A (Sigma Aldrich, St. Louis, MO; 1% in saline) for 30 min. Control mice were anesthetized for 30 min but had no intravesical catheter placement. Pups were returned to their mothers and the treatments repeated twice more on P13/14. Mice were weaned at 3–4 weeks of age and allowed to mature to adulthood (12–14 weeks of age).

### Water Intake Assay

Water intake was measured over a 24-h period prior to assessment of spontaneous voiding. Mice were placed individually in standard polycarbonate mouse cages with food and water *ad libitum*. Water intake was measured in grams as initial weight of water sources minus final weight after 24 h.

### Spontaneous Void Spot Assay

Mice were placed individually in standard polycarbonate mouse cages lined with precut filter paper (Whatman grade 2, cat#1002-320) for a 4-h morning period. Mice had *ad libitum* access to food but water was withheld during the test period. Filter papers were allowed to dry for 24 h and then viewed under ultraviolet light (Spectroline UV-4B ultraviolet lamp) with images of the papers captured electronically and analyzed with the 1.46r version of ImageJ software. Spot area analysis was performed for each urine output spot. Spots of < 6 mm^2^ were used as the lower limit and excluded from analysis to eliminate spots arising from footprints or tail dragging. Results were collected as number of voids and area per void.

### Cystometrograms

Mice were anesthetized with inhaled isoflurane (5% for induction, 3% for maintenance during preparation, and 0.5–0.25% during testing) and 1.2 g/kg urethane IP. Core body temperature was maintained at ∼37°C using a warming pad. A lower midline incision was made and the bladder identified. The bladder was elevated outside of the abdominal wall musculature in order to avoid changes in intravesical pressure due to abdominal contraction, and care was taken not to stretch the bladder pedicle. A small incision was made at the dome and a catheter (PE-60 polyethylene tubing) was inserted. A purse-string 6-0 prolene suture was used to secure the catheter into the bladder, which was kept moist throughout the experiment. Intravesical pressures were measured using an in-line Grass model PT300 Pressure Transducer and data recorded using Spike 2 software (Cambridge Electronic Design, United Kingdom). A syringe pump was used to continuously infuse room temperature saline into the bladder (20 μl/min). Before the infusion was started, the bladder was fully emptied and following initiation of the infusion an initial “threshold” set of measures was obtained for the pressure and volume of distension which first resulted in a micturition response. The bladder function was allowed a 30 min period of infusion to stabilize, then the intercontraction interval (ICI), defined as the time between voiding contractions, was measured during the subsequent 30 min period. An average ICI was calculated for each mouse and used for analysis. Peak contraction pressures were also measured during this 30 min period.

### Adult Bladder Inflammation

Mice were anesthetized with isoflurane (5% induction, 1% maintenance) and a 24-gauge angiocatheter was placed intravesically *via* the urethra following iodine/povidone swabbing of the urethra and administration of ampicillin (50 mg/kg s.c.). Then, 100 μl of zymosan A (Sigma Aldrich, St. Louis, MO; 1% in normal saline) was instilled and allowed to dwell for 30 min. The bladder was then drained and mice were allowed to recover from anesthesia in their home cage. They were then tested 24 hr later for VMRs to UBD as described below. Control mice (no ABI) received all the same treatments except for bladder cannulation and intravesical treatment.

### Acute Foot Shock

Mice underwent a total of six (twice daily × 3 days; 15 min) accommodation sessions with placement in an operant conditioning chamber with a grid floor but received no subsequent stimulation. During a seventh session, mice in the AFS condition received footshock (1 mA, 1 ms) on a variable interval schedule (30 shocks at random intervals in 15 min) while in the chamber. Mice in the control condition (non-footshock; NFS) were treated identically but did not receive AFS on the 7*^th^* session. AFS and NFS mice were placed individually in the same conditioning chambers for each session. Immediately following the final session, mice were anesthetized and underwent measurement of VMRs as described below.

### Visceromotor Responses

Mice received an adult treatment (ABI or AFS) or control treatments as described above. For the VMR measures, mice were anesthetized with isoflurane and oxygen (5% for induction, 3% for maintenance, and 0.25–0.5% during testing) and 1.2 g/kg i.p. urethane. After urethane administration, isoflurane concentrations were lowered until flexion reflexes were present in the hindlimbs, but spontaneous escape behaviors were absent. A 24-gauge angiocatheter was placed intravesically *via* the urethra. Body temperature was maintained at 37°C with a heating pad. Silver wire electrodes were inserted into the left external oblique muscle through an incision in the left abdominal skin. Urinary bladder distensions (UBDs; 20 s) were produced using compressed air and distending pressure monitored using an in-line, low volume pressure transducer. Contractions of abdominal musculature, recorded as EMG activity, were measured *via* the electrodes using standard differential amplification (Grass Inc., P511 AC amplifiers; 50x amplification, 60 Hz clipping, low filter setting 10 Hz–high filter setting 3 kHz) and digitally quantified measures were saved on a computer (Cambridge Experimental Design, Spike 2 software). Electronic rectification of EMG voltage measures to all positive values allowed for calculation of a mean mV measure of the EMG activity during selected time periods such before or during UBD as previously described in rats (e.g., Ness et al., 2021). After a stable anesthesia induction, 60 mmHg distensions (3 min inter-trial) were administered to establish stable (± 20%) responses and were followed by graded, constant-pressure UBDs (20 s duration; 1 min inter-trial interval) at ascending pressures (10–60 mm Hg).

### Statistics

Repeated measures one- and two-way ANOVAs were performed using Systat 12 (SPSS, Inc.; San Jose, CA, United States). Non-parametric analysis was performed using the Wilcoxon-Mann–Whitney *U*-test. Two-tailed probability measures < 0.05 were considered statistically significant. Individual comparisons represent unpaired *t*-tests unless otherwise stated. Data are presented as mean ± SEM unless otherwise described.

## Results

### Spontaneous Voids

**FIGURE 1:**
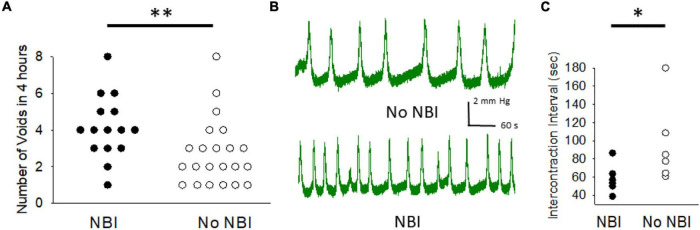
Neonatal bladder inflammation (NBI) results in increased voiding. **(A)** Raw data from 4 h measures of voiding as measured by counting the number of urine spots on filter paper lining holding cage of a single mouse. **(B)** Typical tracings of cystometric measures of intravesical pressure in urethane/isoflurane-anesthetized mice during continuous filling at a rate of 20 μl/minute in mice which had experienced NBI and controls. **(C)** Raw data of mean peak-to-peak intercontraction intervals for mice which had experienced NBI and controls. * and ^**^ indicate statistical difference between NBI and No NBI groups with *P* < 0.05 and *P* < 0.01 (Wilcoxon Mann–Whitney *U* test).

Using the void spot assay, it was determined that adult mice that had experienced NBI had significantly more voids (4.1 ± 0.5; *n* = 15) in the 4 h of measure than mice which had only experienced control treatments as neonates (2.7 ± 0.4; *n* = 22; Wilcoxon–Mann Whitney *U*-test, *p* < 0.005). Figure 1A demonstrates raw data. The presence of increased spontaneous voiding could have reflected increased urine output due to increased water consumption and so this latter variable was also measured in these same mice for 24 h prior to their void spot assay measures. This demonstrated no statistical differences between the two groups with mice that had experienced NBI consuming 3.9 ± 0.3 ml of water and the control mice consuming 3.8 ± 0.3 ml of water (unpaired *t*-test, *p* = 0.8533).

### Cystometrograms

Six mice that experienced NBI were compared with six mice that experienced control treatments. Although intravesical volumes and pressures needed to evoke initial micturition response were lower in the NBI group compared with controls (15 ± 1 vs. 20 ± 3 μl and 4.8 ± 1.1 vs. 5.2 ± 0.4 mm Hg, respectively), these differences were not statistically significant. However, with continuous infusion of saline intravesically at a rate of 20 μl/min, repetitive contractions occurred in both groups (examples in Figure 1B) with a statistically lower ICI of 58.6 ± 6.5 s in the NBI group versus 96.4 ± 18.6 s in the control group (raw data in Figure 1C; Wilcoxon–Mann–Whitney *U*-test, *p* < 0.05). Peak pressures of contraction were comparable (9.4 ± 1.5 vs. 9.5 ± 0.6 mm Hg) between the two groups.

### VMRs Following ABI

**FIGURE 2:**
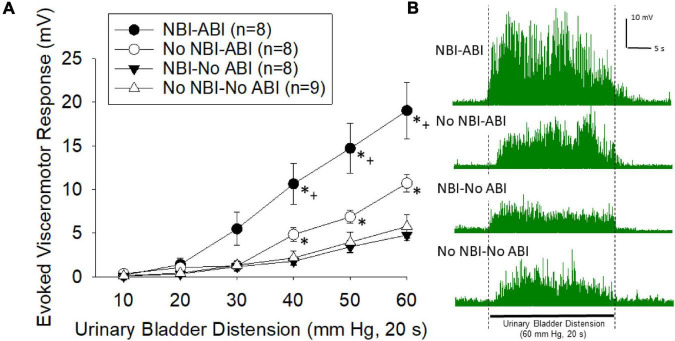
**(A)** Graphical presentation of mean evoked visceromotor responses in mice which had received neonatal bladder inflammation (NBI) and adult bladder re-inflammation (ABI) and their respective controls. Evoked response consisted of rectified mean voltages of electromyographic (EMG) activity during urinary bladder distension (UBD) minus the mean EMG activity immediately prior to UBD. Responses were statistically greatest in the NBI-ABI group. **(B)** Typical examples of rectified EMG activity of individual mice (from groups as labeled) during a 60 mm Hg, 20 s UBD. For statistical analysis, see text. * indicates statistically greater response than both No-ABI groups (*p* < 0.05), + indicates statistically greater response than the No NBI-ABI group (*p* < 0.05).

Visceromotor responses following adult bladder inflammation were more vigorous in mice which had experienced NBI than in control mice (overall repeated measures ANOVA *F*_3,29_ = 11.319; *p* < 0.001; Figure 2A, example traces in Figure 2B). The responses of mice that did not experience ABI were not different when compared according to their neonatal treatments (NBI-no ABI vs. No NBI-No ABI group comparison generated *F*_1,15_ = 0.267). However, there was an effect of ABI in both the NBI and control groups (NBI-ABI vs. NBI-no ABI groups *F*_1,14_ = 16.104, *p* < 0.001; No NBI-ABI versus No NBI-No ABI group comparison *F*_1,15_ = 5.501, *p* < 0.05). Comparison of the NBI-ABI versus the No NBI-ABI group demonstrated the former group to be statistically most robust (*F*_1,14_ = 6.676; *p* < 0.05). Individual *post hoc* comparisons for individual intensities of UBD are presented in Figure 2.

### VMRs Following AFS

**FIGURE 3:**
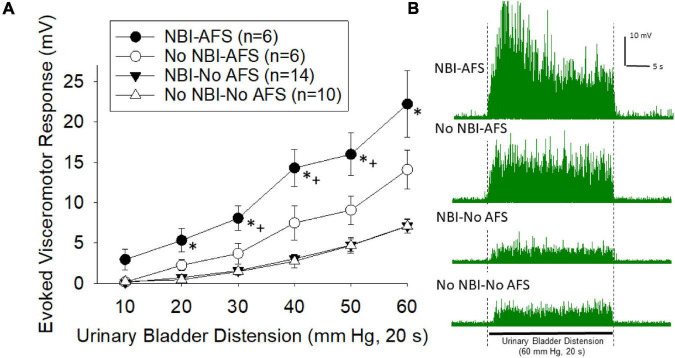
**(A)** Graphical presentation of mean evoked visceromotor responses in mice which had received neonatal bladder inflammation (NBI) and adult acute footshock (AFS) and their respective controls. Evoked response consisted of rectified mean voltages of electromyographic (EMG) activity during urinary bladder distension (UBD) minus the mean EMG activity immediately prior to UBD. Responses were statistically greatest in the NBI-AFS group. **(B)** Typical examples of rectified EMG activity of individual mice (from groups as labeled) during a 60 mm Hg, 20 s UBD. For statistical analysis, see text. * indicates statistically greater response than both No-AFS groups (*p* < 0.05), + indicates statistically greater response than the No NBI-AFS group (*p* < 0.05).

Visceromotor responses following acute footshock were more vigorous in mice which had experienced NBI than in control mice (overall repeated measures ANOVA *F*_3,32_ = 20.416; *p* < 0.001; Figure 3A, examples in Figure 3B). The responses of mice which did not experience AFS were not different when compared according to their neonatal treatments (NBI-no AFS vs. No NBI-No AFS group comparison generated *F*_1,22_ = 0.90; n.s.). However, there was an effect of AFS in both the NBI and control groups relative to their NFS counterparts (NBI-AFS vs. NBI-no AFS groups *F*_1,18_ = 44.493, *p* < 0.001; No NBI-AFS versus No NBI-No AFS group comparison *F*_1,14_ = 8.762, *p* = 0.010). Comparison of the NBI-AFS versus the No NBI-AFS group demonstrated the former group to be statistically most robust (*F*_1,10_ = 5.664; *p* < 0.05). Individual *post hoc* comparisons for individual intensities of UBD are presented in Figure 3.

## Discussion

The most important finding of the present study was that mice that experienced NBI were hypersensitive to urinary bladder filling as adults. This was observed as an increase in the number of spontaneous voids, an increase in the number of voids evoked by active bladder filling, and by increased vigor in the VMRs to UBD when a secondary insult (ABI or AFS) was introduced. Similar findings have been demonstrated in rats treated with NBI (Ness et al., 2021) and these are the clinical features of IC/BPS. As noted in the Introduction, many behavioral, neurophysiological and neurochemical measures are altered in adult rats which have experienced NBI. The ability to use mice in this model system will allow significant methodological expansion and probing of mechanistic questions due to the extensive genetic and immunological manipulations that are possible in murine systems. A recent scientific panel of the NIH-sponsored multicenter Multidisciplinary Approach to Chronic Pelvic Pain (MAPP) Network (Lai et al., 2015) identified that non-human animal models of IC/BPS are still needed since there are limitations to the models currently in use which they reviewed. These included bladder inflammation due to acute bacterial infections, systemic cyclophosphamide, autoimmune reactions to ovalbumin, MCP-1 reactions that occur in response to pseudorabies injections, and acute/chronic stress models. Despite the extensive nature of previous studies, the changes in neurophysiology and neurochemistry that occur in subjects with IC/BPS have still not been determined. Research using other visceral nociceptive systems suggest there could be a role for novel interactions with corticotrophin-releasing factor receptor (CRFR) systems (e.g., Fuentes and Christianson, 2018), but definitive evidence for a role of any receptor will likely need use of related genetic models to probe the role of those receptors. Autoimmune phenomena have also been implicated as etiologies of IC/BPS and so availability of murine immunobiologic models will similarly allow probing into those potential mechanisms.

There exist epidemiological correlates that support potential mechanisms associated with the NBI model. Namely, childhood bladder infections with secondary inflammation are common in females, occurring approximately 2–10 times more frequently than in males during preschool and early grade school years (Tullus and Shaikh, 2020). Notably, females are similarly 2–10 times more likely to develop IC/BPS than males (Hand, 1949; Koziol et al., 1993; Jones and Nyberg, 1997; Bogart and Clemens, 2007; Clemens et al., 2019). Retrospective epidemiological surveys indicate that urinary tract infections are more common in women who subsequently develop IC/BPS than in non-IC/BPS women (Peters et al., 2009). Obviously, such correlations do not imply causation, and most individuals who experience a urinary tract infection as a child do not develop IC/BPS. Based on these observations, we postulate that the development of IC/BPS in at least a subset of individuals is due to a childhood “priming” event and an adult “second insult” which could have multiple etiologies. Such a two insult mechanism might explain the difficulties which epidemiologists have encountered when attempting to determine a single cause for IC/BPS.

Studies in rats support that phenotypic changes in adults only manifest if the NBI event occurs during a critical period of development. Specifically, the intravesical administration of zymosan results in augmented adult responses only if it occurs in the period of time that would correspond with late neonatal-early childhood period (in rats P14-P16). Testing of NBI at other periods of development (P7-10, P21-23, P28-30, P90-92) have all failed to elicit the responses noted with NBI on P14-16 (Randich et al., 2006; Deberry et al., 2007; Ness et al., 2021). Based on studies by Fitzgerald (2005), this critical time period is when the central nervous system is developing descending modulatory circuits and the organism is “switching” from a neonatal phenotype to an adult form. The P12-14 dates chosen for treatment of mice in the present study were selected to be in this same window of development for mice. Studies in rats have suggested that there may be a failure in the feedback inhibitory systems activated by acute inflammatory events in NBI rats (Deberry et al., 2007), in particular in opioidergic systems (Deberry et al., 2007; Shaffer et al., 2013a,b), however, alterations in GABAergic and glycinergic systems have also been identified (Ness et al., 2018).

The present study also gives evidence that NBI leads to increased urinary frequency without a secondary insult as evidenced by a shortened intercontraction interval when a continuous intravesical infusion is administered. Initial cystometric volume and pressure threshold measures were not statistically different in the NBI versus control groups, but this may represent the small sample size (6 per group), as well as the surgical procedure needed for these measures which involves cannulation of the dome of the mouse bladder. This surgical manipulation alters the structure of the bladder itself and so could have obvious impact on such measures and their variability. Once stable contractions of the bladder were established using a continuous infusion of saline, it became apparent that the mice which had experienced NBI had bladders which were more responsive to distension. This was consistent with the increased number of spontaneous voids in mice which had experienced NBI.

Nociceptive responses to UBD, quantified as VMRs, were most robust in mice that had experienced NBI. It is notable, however, that the exaggerated nociceptive visceromotor reflex responses to UBD only became manifest following a secondary insult—in this case, ABI or AFS. In rats, we have demonstrated that other pain-related manipulations can also result in augmented VMRs to UBD. This includes somatic inflammation or neuropathic injury that can be either hetero- or homo-segmental (Ness et al., 2021). It will be possible to probe mechanisms of this augmentation of nociceptive responses in mice using the present model.

In conclusion, a murine model of bladder hypersensitivity produced by NBI has been described. Given the potential importance of NBI as part of the pathophysiology associated with IC/BPS and the similarity of the mouse and rat phenotypes to clinical signs/symptoms of the disorder, this model system will allow for a probing of multiple mechanisms using targeted strategies currently unavailable in the rat model that may lead to novel therapeutics in humans.

## Data Availability Statement

The raw data supporting the conclusions of this article will be made available by the authors, without undue reservation.

## Ethics Statement

The animal study was reviewed and approved by Institutional Animal Care and Use Committee at the University of Alabama at Birmingham.

## Author Contributions

BC-M and TN contributed to the data collection and analysis. JD and TN contributed to study design. All authors have assisted in the generation and revision of this manuscript and have read and approved the final version of the manuscript.

## Conflict of Interest

The authors declare that the research was conducted in the absence of any commercial or financial relationships that could be construed as a potential conflict of interest.

## Publisher’s Note

All claims expressed in this article are solely those of the authors and do not necessarily represent those of their affiliated organizations, or those of the publisher, the editors and the reviewers. Any product that may be evaluated in this article, or claim that may be made by its manufacturer, is not guaranteed or endorsed by the publisher.

## Abbreviations

ABI: acute bladder inflammation
AFS: acute footshock
AUC: area-under-the-curve
EMG: electromyogram
IC/BPS: interstitial cystitis/bladder pain syndrome
IP: intraperitoneal
IT: intrathecal
NBI: neonatal bladder inflammation
NFS: non-footshock
UBD: urinary bladder distension
VMR: visceromotor response.
